# Silica diatom shells tailored with Au nanoparticles enable sensitive analysis of molecules for biological, safety and environment applications

**DOI:** 10.1186/s11671-018-2507-4

**Published:** 2018-04-10

**Authors:** V. Onesto, M. Villani, M. L. Coluccio, R. Majewska, A. Alabastri, E. Battista, A. Schirato, D. Calestani, N. Coppedé, M. Cesarelli, F. Amato, E. Di Fabrizio, F. Gentile

**Affiliations:** 1 Department of Experimental and Clinical Medicine, University of Magna Graecia, 88100 Catanzaro, Italy; 2 IMEM-CNR, Parco Area delle Scienze 37/A, 43124 Parma, Italy; 3 Unit for Environmental Sciences and Management, School of Biological Sciences, North-West University, Potchefstroom, 2520 South Africa; 4 South African Institute for Aquatic Biodiversity, Grahamstown, 6140 South Africa; 5 Department of Physics and Astronomy, Rice University, Houston, TX 77005 USA; 6 Interdisciplinary Research Center on Biomaterials, University Federico II, 80125 Naples, Italy; 7 Department of Physics, Politecnico di Milano, 20133 Milan, Italy; 8 Department of Electrical Engineering and Information Technology, University Federico II, 80125 Naples, Italy; 9 King Abdullah University of Science and Technology, Thuwal, 23955-6900 Saudi Arabia

**Keywords:** Diatoms, Frustules, Sensing devices, Au functionalization, SERS, Safety, Biological sensing

## Abstract

Diatom shells are a natural, theoretically unlimited material composed of silicon dioxide, with regular patterns of pores penetrating through their surface. For their characteristics, diatom shells show promise to be used as low cost, highly efficient drug carriers, sensor devices or other micro-devices. Here, we demonstrate diatom shells functionalized with gold nanoparticles for the harvesting and detection of biological analytes (bovine serum albumin—BSA) and chemical pollutants (mineral oil) in low abundance ranges, for applications in bioengineering, medicine, safety, and pollution monitoring.

**Electronic supplementary material:**

The online version of this article (10.1186/s11671-018-2507-4) contains supplementary material, which is available to authorized users.

## Background

Diatoms are unicellular algae massively present on earth with over 100,00 species distributed in aquatic (oceans, lakes, rivers) and semi-aquatic (wetlands and soils) niches. They contribute to an estimated 40–50% of the total organic material content in the oceans and to the ~ 20% of the conversion of carbon dioxide to organic compounds (i.e., photosynthesis) in the biosphere [1–3].

Diatoms are protected by a functional silicon dioxide shell (frustules) with a complex, micrometer-scale architecture and a pore size varying between species. Due to their microstructure, diatom shells show values of specific strength up to ~ 1700 kN m/kg that lay well above those of other natural cellular, composite, and silk materials including spider silk (1000 kN m/kg) [4–7]. Moreover, due to the regularity and symmetry in the lattice of pores aligned on the frustule surface, often diatom frames show natural optical properties, and reveal light convergence, concentration, and trapping effects, depending on pore geometry and topology, wavelength, and the valve orientation [8–12].

Thus, diatoms are natural (in opposition to artificial), abundant, low cost, and easily accessible three-dimensional micro- or nanoscale structures that do not require traditional techniques of nanofabrication for their production, and, in sight of their scale, morphology, and properties thereof, display potential for being utilized as miniature sensors, drug-delivery capsules, and other micro-devices [2, 13, 14]. Nevertheless, despite this promise, there are relatively few applications of diatoms in nanotechnology [15–17], possibly because, while diatom shells represents the required support for many structures, further functionalizations (modifications) are necessary to provide these structures with the correct functions.

**Fig. 1:**
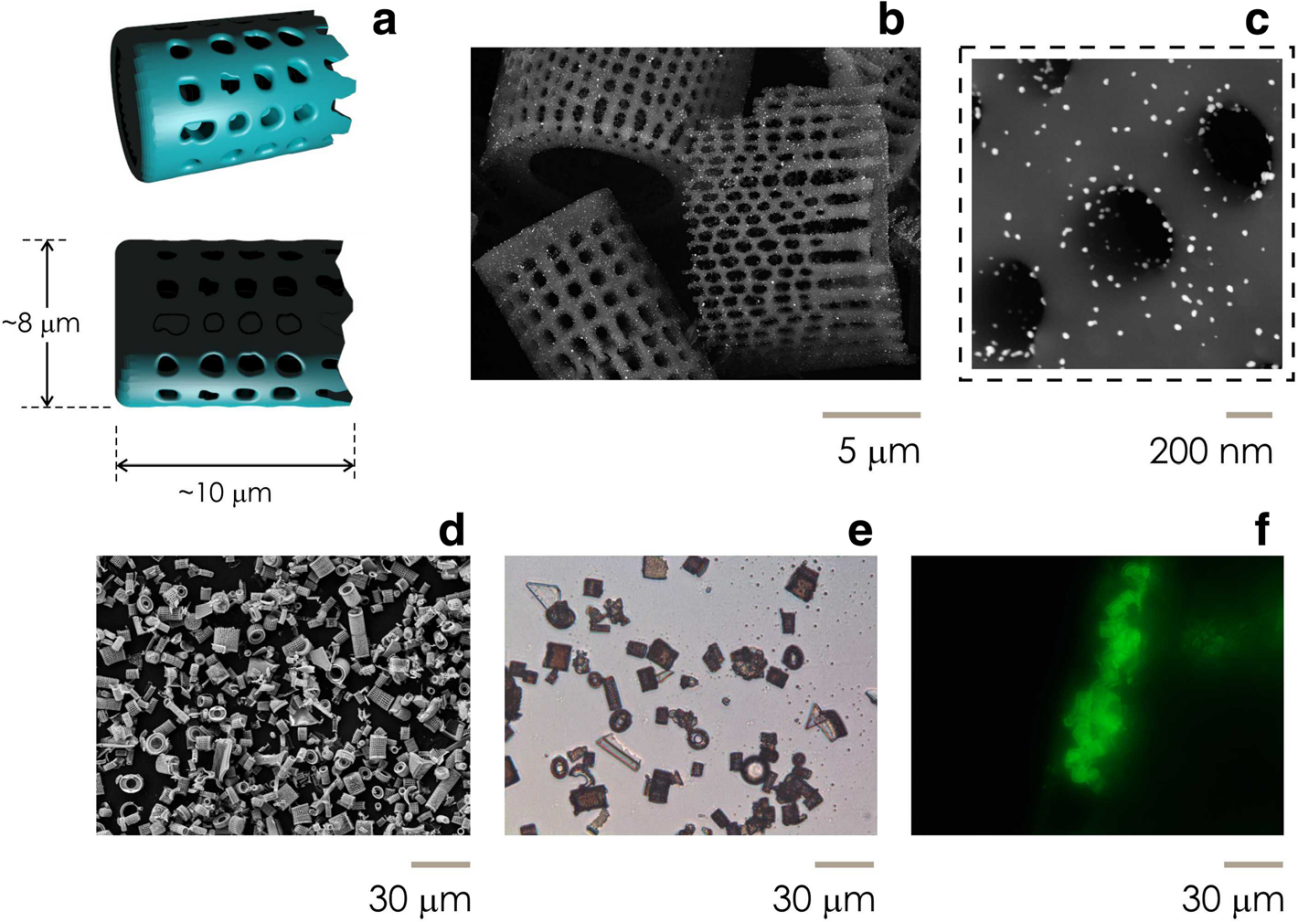
Artist’s impression of silicon dioxide diatom shells, which appear as micrometer cylinders with an average diameter of *d*~8 μm and heights larger than *h* > 10 μm, arrays of pores decorate the external surface of the diatoms (**a**). SEM micro-graphs of silica shells functionalized with gold nanoparticles (D24 systems) acquired at low (**b**) and high (**c**) magnifications factors. From these, one can observe the regular pattern of pores permeating the diatom surface decorated with randomly distributed gold nanoparticles, with a pore size ~200 nm and a particle size ~20 nm. Large field SEM (**d**) and optical (**e**) images of D24 systems assess the functionalization process capability to produce large volumes of micro-devices. Fluorescence microscopy inspection of D24 systems after incubation with fluorescent 50 nm yellow microspheres reveal devices selectivity, specificity and sensitivity (**f**)

In this *letter*, we demonstrate a method to functionalize silica diatoms shells with Au nanoparticles. This results in devices with multiple scales in a hierarchical design. Each shell is a silicon dioxide cylinder with an average diameter *d* ~ 8 μm and height *h* ~ 10 μm (Fig. 1a and Additional file 1). Shell surfaces incorporate dense patterns of pores that are approximately circular in shape and their size varies in the narrow interval *p*_*s*_ = 200 ± 40 nm (Fig. 1b, c). Gold nanoparticles are then distributed uniformly over the external surface of the shells, with an average diameter of the particles of *Au* − *np*_*s*_~20 nm and small deviations around the mean (Fig. 1b, c). Since diatom shells are here derived from diatomaceous earth, that is a low cost, theoretically unlimited source of frustule (Additional file 2), the method yields high volumes of nano-devices in a small time (Fig. 1d, e).

The device integrates different scales. (i) The sub-millimeter dimension of the shells permits system manipulation, handling, and access. (ii) The micrometer size of the pores enables molecule-harvesting, selection, and (in a more sophisticated evolutions of the device) fragmentation. (iii) The nanometer size of the Au–NPs enables control and amplification of an external electromagnetic (EM) radiation. Thus a hierarchical multi-scale architecture permits to extract-specific analytical molecular targets from a solution and characterize them using surface-enhanced Raman spectroscopy (SERS) even in very low abundance ranges. Incubation with fluorescent 50 nm microspheres (Fluoresbrite® Yellow Green Microspheres—Additional file 3) and subsequent fluorescence analysis indicates devices localization, selectivity, specificity, and absence of signal from the background (noise) (Fig. 1f).

## Results

### Functionalization with Au–NPs

**Fig. 2:**
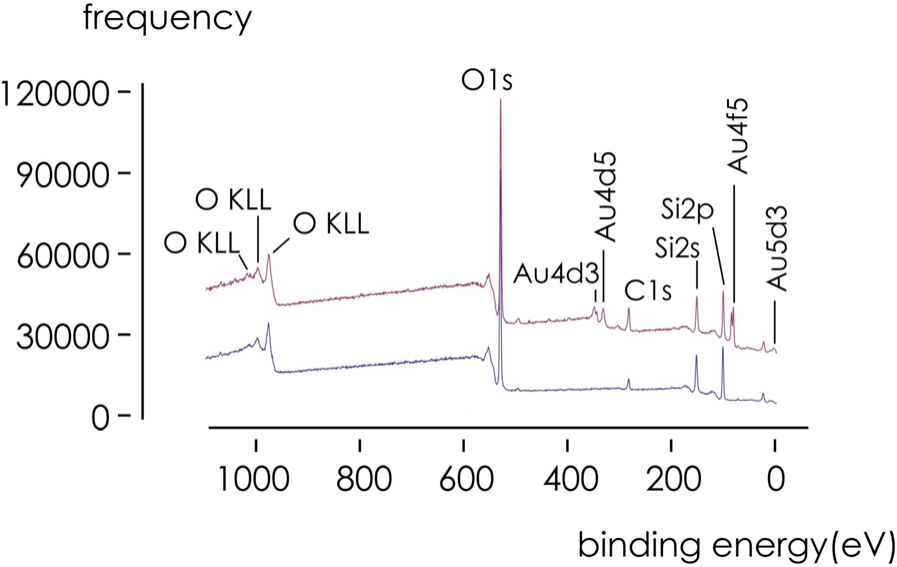
XPS spectra of silicon dioxide diatom shell before (lower diagram) and after (upper diagram) functionalization with gold nanoparticles

Diatomaceous earth (DE) was cleaned with piranha solution to remove organic residuals. Samples were then maintained for 120 s in a diluted 2% hydrofluoric acid (HF) solution to remove small fragments, roughen diatoms surface, and promote Au nucleation. Samples where then decorated with Au–NPs using of a photo-deposition process. Shells were suspended in DI water with 0.1% solution of chloroauric acid (HAuCl_4_) in isopropyl alcohol and illuminated with a UVA/UVB Osram Ultra Vitalux lamp. Irradiation time, concentration of diatom shells in solution, and amount of chloroauric acid were varied over significant intervals to produce different nanoparticles morphologies. For the present configuration, we used 20 mg of shells in 50 ml of solvent and timely injections of 30 μl of chloroauric acid every 5 min, for a total duration of 1 h. Notice that the method does not imply electrochemical reduction of gold ions into metallic gold as in electroless deposition [18, 19]. In the following, we shall indicate Au–NPs functionalized diatom shells with the abbreviation D24. X-ray photoelectron spectroscopy (XPS) was used to characterize D24 systems. High-resolution XPS spectra were acquired using a power *P* = 100 W, beam energy *e* = 11.7 keV, resolution *δe* = 0.1 eV, accumulation time of *t* = 20 min minimum. Peaks in the spectra were referenced to the carbon peak C1s at 284.8 ev binding energy. In Fig. 2, we report XPS spectra of systems before and after functionalization. We observe that, after functionalization, D24 systems display the emergence of metallic gold (core band Au4f5 at 84 ev binding energy), and traces of the valence bands associated to Au4d3 (353 eV), Au4d5 (334 eV), Au5d3 (6 eV). We also observe traces of Sodium (Na1s, 1071 eV and Auger peaks at 497 eV) and Silicon (Si2s, 2p at 150 and 97 eV), that are attributed to contaminants in the substrate used for nanoparticles drop deposition. Presence of carbon (C1s) in the spectra is coincidental, not associated to the fabrication process, and it results from the spontaneous adsorption of normal levels of carbon contained in the atmosphere to the diatom surface. The presented method for the synthesis of nanoparticles enables the formation of nanoparticles on the external surface of the diatoms and within the pores. Additional scanning electron microscopy (SEM) images presented in Additional file 1 demonstrate deposition of Au nanoparticle deep inside the pore matrix of the diatoms. So, while pores in the porous matrix allow analytes sequestration, immobilization, and retention, gold nanoparticle array enable SERS effect and the detection of analytes in very low abundance ranges. These effects are tightly interwoven.

### Simulating EM Field Around D24/Au–NPs

**Fig. 3:**
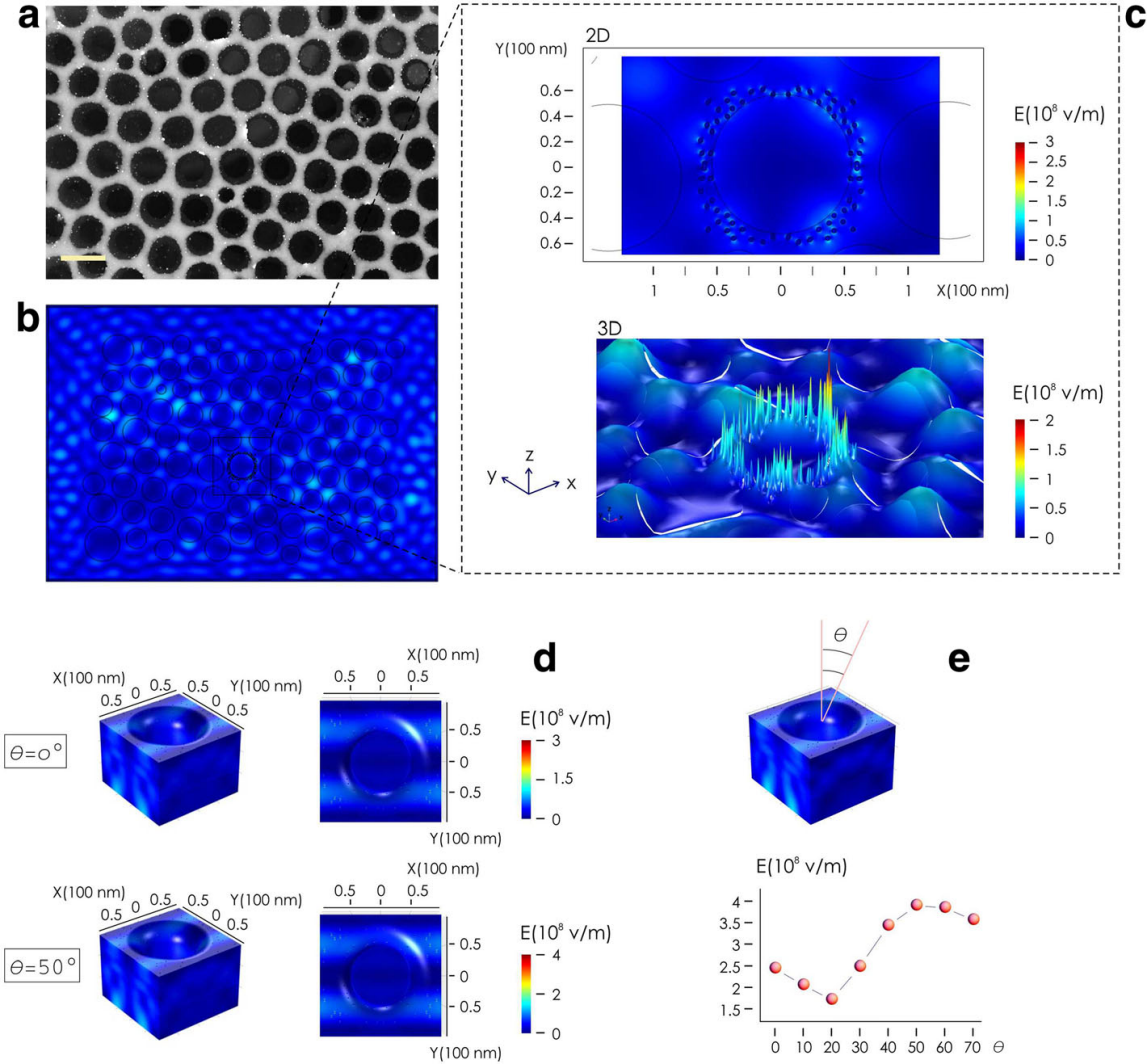
SEM image of an hexagonal lattice of pores on the diatom surface reproducing a photonic crystal (**a**). Pore size, shape, and topology of the real prototype, and random patterns of gold NPs distributed between the pores, were replicated in a numerical finite element analysis (FEA) tool-box (**b**). The output of the simulations is the EM field and the EM field enhancement around the aggregates of gold nanoparticles, with a maximum EM field of nearly ~3 10^8^ V/m (**c**). EM distribution shows sensitivity to the orientation of the pore surface with respect to the external incident radiation (**d**). Varying the angle of incidence between the external radiation and the normal of the pore surface over significant intervals, we find that the maximum EM field intensity oscillates between ~1.5 10^8^ and ~4 10^8^ V/m (**e**)

We used computer simulations and finite elements analysis (FEA) to evaluate the EM field around arrays of gold nanoparticles in the D24 system (Methods and Additional file 4). Since patterns of holes in the diatom reveal an hexagonal symmetry (Fig. 3a) resembling a photonics crystal, we used a numerical scheme to evaluate whether a similar geometry, decorated with a uniform distribution of Au nanoparticles, may locally enhance the EM signal. Simulated patterns of pores were reproduced from a real SEM image (Fig. 3b). A maximum of 125 particles were placed around each pore and between the pores (Fig. 3b). We approximated the incident EM field with a TM linearly polarized plane wave with central wavelength *λ* = 633 nm, power *P*_*inc*_ = 1 W, and associated power density *I* = 2.5 × 10^8^ W/cm^2^. In the simulations, diatom shell was described by a dielectric with refractive index *n*_D24_ = 1.3 and the surrounding medium and the pores were considered air with *n*_air_ = 1. Au–NPs were modeled using the formulation of Rakic and colleagues [20]. Results indicate (Fig. 3c) that the EM field amplified by the system is unevenly distributed in the volume of interest, with the EM field preferentially concentrated around the gold nano-particles, where it achieves intensities as high as |E|~3 × 10^8^ V/m, and associated enhancement factors Q~10^2^ if we consider the EM field and Q~10^8^ if we consider the surface enhanced Raman spectroscopy (SERS) effects. (In which case, the enhancement is proportional to the amplitude of local electric field to the power of four [21]). Since in practical applications diatom surface may by randomly orientated with respect to an external radiation, it has some interest analyzing the behaviour of |E| as a function of the direction θ that the normal of the diatom surface forms with the propagating TM wave (Fig. 3e). In the θ = 0 − 70^°^ interval, |E| oscillates between ~1.5 × 10^8^ V/m = |E|_min_ at θ = 20^°^ and ~4 × 10^8^ V/m = |E|_max_ at θ = 50^°^. Thus the intensity of the EM field is severely influenced by the way in which D24 systems are positioned on a surface for successive analysis and inspection. Notice however that even in the worst configuration, calculated values of |E|_min_ are sufficiently large to yield a robust and sensitive analysis of the signal associated to the propagation of the EM field.

FEM analysis presented in Fig. 3a–c simulates the EM field in simplified 2D planar geometries—for this configuration, the EM field evolves around the Au nanoparticles on the external diatom surface. Nevertheless, in the three-dimensional scheme of Fig. 3d and other images presented in the Additional file 4, gold nanoparticles are distributed along the internal surface of the pores. This scheme, more akin to the real physical prototype, indicates that the analytes adsorbed by the D24 devices may interact with the EM field—and being detected—for any pore/analyte reciprocal localization. So, even if SERS is a short range effect and the EM field decays with a power of three of the distance from the gold nanoparticles [22], analyte harvesting and analyte/pore colocalization assure the sensing capabilities of the device. Further to this end, we demonstrate localization of the analyte in the pores with additional, high magnification fluorescence images of D24 systems loaded with Yellow Green 50 nm nanospheres (Additional file 3). Spatial overlap between the fluorescence signal and the D24 diatom devices and the vanishingly small background signal demonstrate that analyte uptake is highly efficient leaving no or minimal residues.

### SERS Analysis of BSA in Solution

Here, we assess the ability of D24 devices to operate as molecular harvesting agents and sensing devices in biological systems. We incubated D24 devices in a solution containing bovine serum albumin (BSA) in a 10^−16^ M concentration, with a relative abundance of 1 mg of D24 devices in 1 ml of solution. The complex network of openings that penetrates into the diatom represents a filter that can absorb the molecules with a hydrodynamic diameter smaller than the pore size. Considering that, for the present configuration, the average pore size is nearly 200 nm, BSA proteins with a characteristic length size of ~ 6 nm [21] would easily accumulate within the pore matrix. After 10 min from incubation, D24 systems were separated and extracted from the initial solution through sedimentation. D24 devices containing BSA were positioned on the stage of a Renishaw inVia micro-Raman microscope for analysis.

**Fig. 4:**
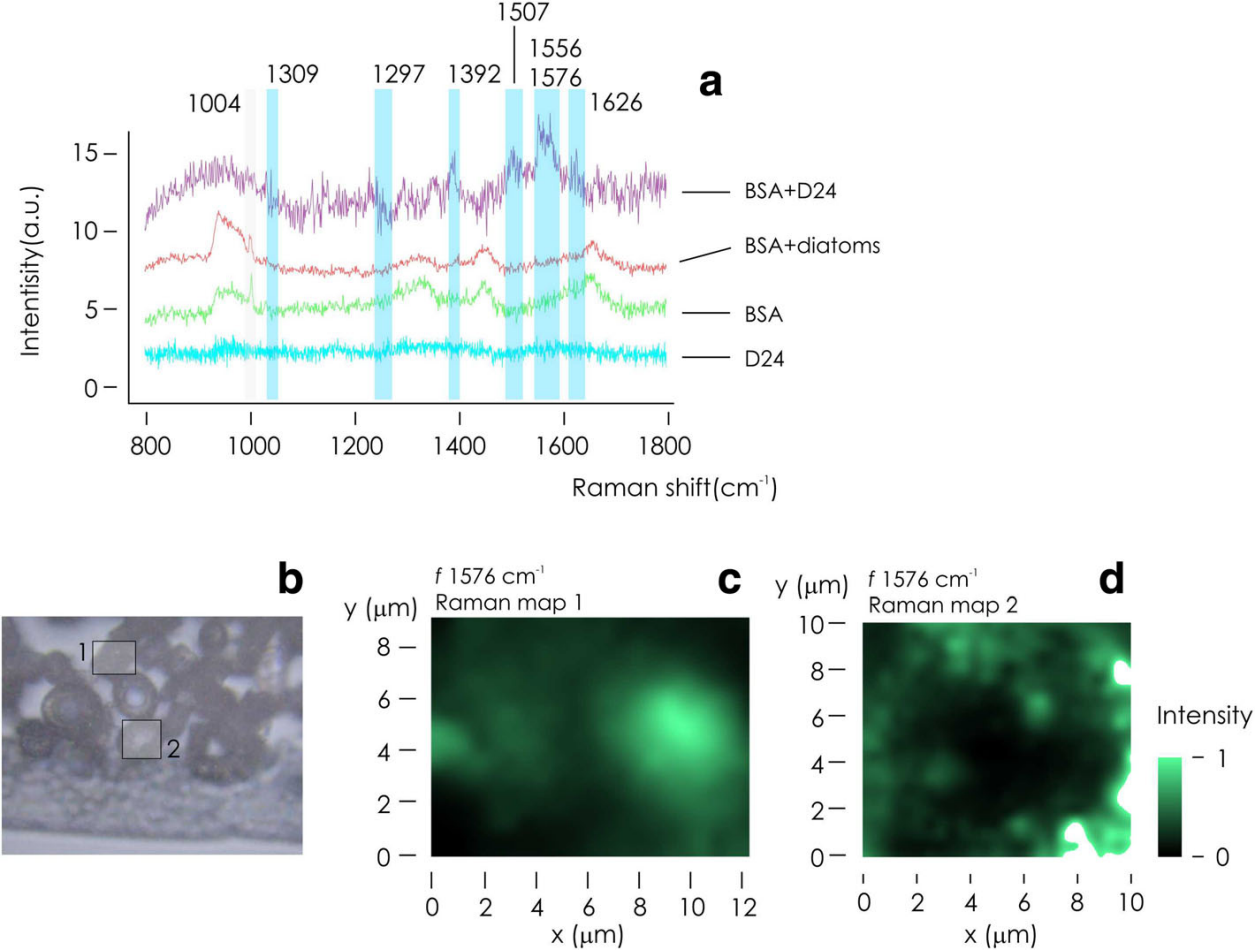
Raman spectra of pure BSA, BSA adsorbed by silicon dioxide shells, and BSA adsorbed by D24 systems, in the latter two experiments, the initial concentration of BSA was 10^−16^ (**a**). Optical microscopy inspection of D24 systems after incubation with BSA (**b**); Raman map of BSA acquired over individual D24 systems (**c**, **d**)

Figure 4a reports the measured Raman spectra of the D24 capsule (i), of pure BSA (ii), of BSA + non-functionalized diatom shells (iii), and of BSA + D24 systems (iv). Notice that in the last configuration, the systems yield SERS effects. In Table 1**,** we report a direct comparison and a tentative assignment of peaks measured in the systems with (iii) and without (ii) SERS effects. While BSA is still detectable in simple diatom shells, Au nanoparticles in D24 systems highlight the presence of the aromatic components of BSA at 1392 cm^−1^ and in the 1556–1576 cm^−1^ band. The peak at 1670 cm^−1^ is suggestive of the presence of Amide I in the sample, that in turn suggests *β*- sheet conformation visible with SERS. The corresponding peak in simple micro Raman is located at 1658 cm^−1^, that differently suggests an *α*-helix structure. At the same time, the relevant enhancement of the COO- symmetric stretching at 1392 cm^−1^ suggests strong electrostatic interactions with the diatom/gold surface [23]. SERS matrix scans of samples over finite areas were carried out at the central frequency *f* = 1576 cm^−1^ to assess reparability, reliability, and sensitiveness of the measurements (Fig. 4b). Raster plot of SERS signal in two different configurations (Fig. 4c, d) indicate the system capability of reconstructing the spatial distribution of BSA content across shells in vanishingly low abundance ranges. In previously reported experiments [24], we have investigated local heating induced by EM amplification and plasmonics. While we observed relevant and site-selective temperature increments associated to nano-photonics devices on a substrate, with absolute values of temperatures as high as ~ 400 K, nevertheless laser power associated to these increments should be set in the 10 mW range, i.e., two orders of magnitude higher than laser power intensity *P* = 0.18 mW used for current measurements. Therefore, in this case, artificial heating effects and possible conformational changes in proteins are neglected. Conformational changes and changes in the relative content of *β*- sheets in proteins are activated by externally applied temperature fields starting from ~ 340 K [25].

**Table 1:** Raman and SERS peaks of BSA in the 800–1800 cm^−1^ range, and tentative assignment of peaks

Raman peak BSA	SERS peak BSA	Tentative assignment
1004	1004/1039	Phenylalanine (Phe) ring breathing
	1228–1297	Amide III [26–28]
1325		CH_2_ twisting [27] orCH bending [28]
1401	1392	Aromatic amino acids COO- stretching [27–29]
1450	1450	CH_2_ and CH_3_ scissoring [26–28]
	1507	Carbon skeleton stretching modes at 1509 cm^−1^
	1556–1576	Trp - Phe
	1626	Tyr + Phe [27, 28]
1658	1670	Amide I [27, 28]

### SERS Analysis of Mineral Oil

D24 devices were demonstrated in the analysis and detection of mineral oil in increasingly low dilution factors. Mineral oil is a by-product of the distillation of petroleum to produce gasoline. It contains light mixtures of higher alkanes with the paraffins of this mineral oil ranging from about *C*_18_ to *C*_40_: it roughly corresponds to the composition of base oil for manufacturing lubricating or hydraulic oils [26]. In a recent commentary [26], it is recommended to reduce the exposition to mineral oil to levels lower than ~ 50 mg/kg, i.e., 50 ppm. Analysis of mineral oil (m.o.) and related products is therefore of interest in environmental pollution and food safety. m.o was examined using Raman spectroscopy following the methods described in the previous BSA analysis.

**Fig. 5:**
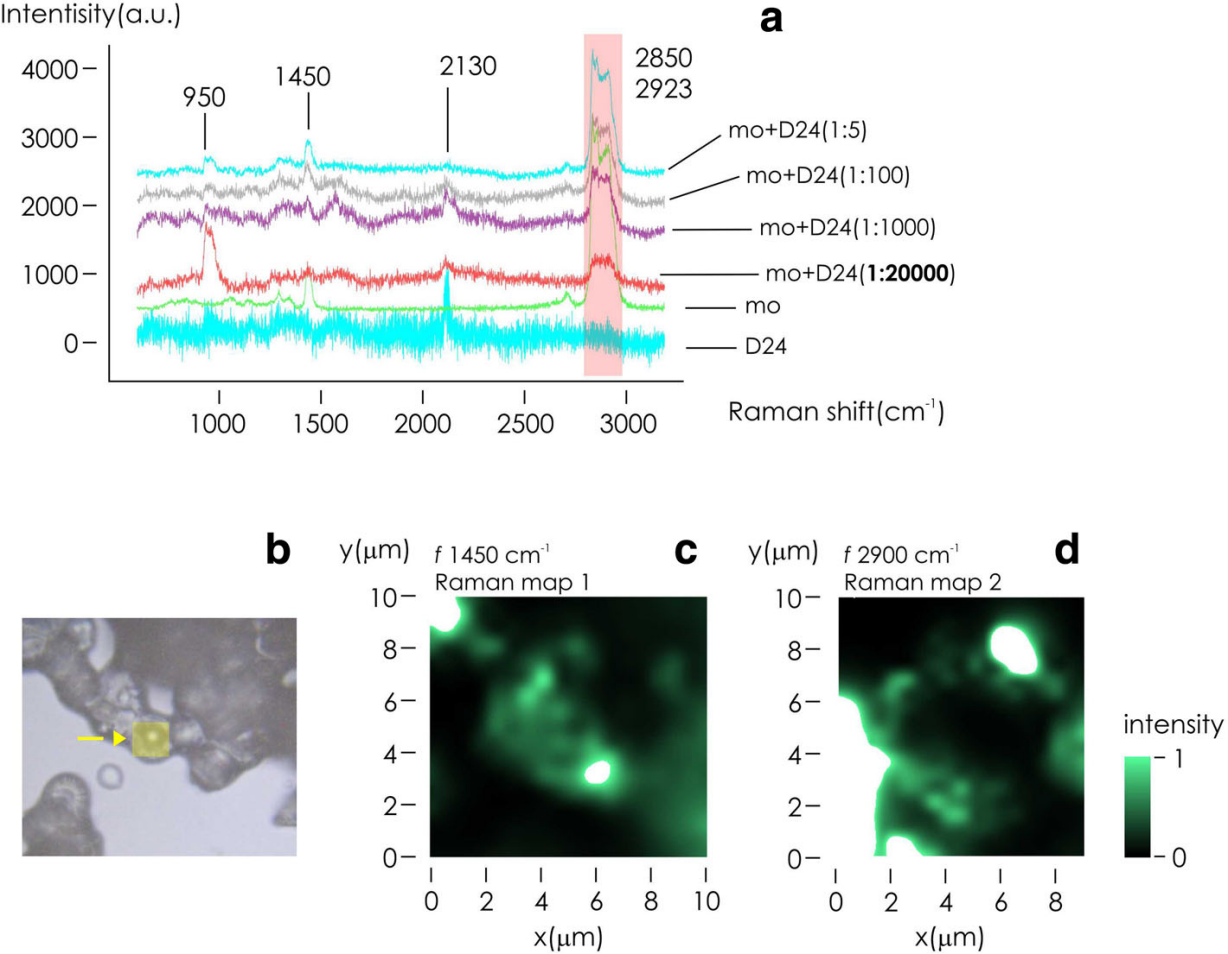
Raman spectra of D24 systems, pure mineral oil, and mineral oil adsorbed by D24 systems in increasingly low concentrations (**a**). Optical image of D24 systems after incubation with mineral oil and sedimentation (**b**). Raman maps of mineral oil adsorbed by a D24 micro device acquired at *f* = 1450 cm^−1^ (**c**) and *f* = 2900 cm^−1^ (**d**)

Figure 5a displays the measured Raman spectra relative to the sole D24 capsules (i), to the sole m.o. (ii), and to an emulsion of m.o. and DI water in different concentrations (iii). Silicon dioxide is the principal component of the D24 systems (i). In the considered range of frequencies 600–3200 cm^−1^, we observe Raman peaks in the band 950 cm^−1^, associated to a second-order scattering of Silicon, and in the band 2130 cm^−1^, associated to the −SiH_2_ stretching [27]. The m.o*.* spectrum (ii) is characterized by the peak at 1450 cm^−1^, indicative of the CH_2_ scissors vibration, and the peaks in the 2850–2923 cm^−1^ region, attributable to CH stretching [28]. Raman spectra relative to D24 after adsorption with m.o. at different concentration ranging from 0.05 to 200 μl/ml indicate that the relative content of m.o. in the emulsion is encoded in the bands 1450 and 2850–2923 cm^−1^. The higher the content of m.o. in the emulsion, the higher the Raman peaks in these frequency ranges. Remarkably, D24 analysis is sensitive to m.o. dilutions as low as 0.050 μl/ml ≡ 50 ppm, (m. o. : DI water), that is the threshold limit value above which safety, toxicity, or pollution concerns may rise. Raman maps of m.o. at a dilution of 10 μl/ml are measured over a D24 surface and reported in the inset of Fig. 5b. The maps are calculated at the central frequencies *f* = 1450 cm^−1^ (Fig. 5c) and *f* = 2900 cm^−1^ (Fig. 5d). In all cases, Raman intensity is proportional to the content of m.o. in the diatom shell, and the m.o. profile is reconstructed with sub-micrometer resolution.

## Discussion

The described scheme allows analyte capture, localization, sequestration, and detection. Analytes adsorbed by porous D24 diatoms can be easily collected, manipulated, separated, and aliquoted into samples. Each sample is constituted by (i) functionalized diatoms loaded with (ii) specific analytes. Thus a sample is combination of the analyte and the device necessary to detect it. Different aliquots can be processed using simple Raman set-ups, stored for future analysis, perhaps kept in a refrigerator or a freezer for long periods of time. Thus, D24 systems are a hybrid device which operates in symbiosis with the target molecules that one wants to analyze. While traditional SERS substrates or metal nanoparticles have been heretofore used in isolation, and interaction between the SERS substrate and the analyte takes place in intermittent episodes and is often limited at the time of the measurement, D24 systems integrate the sensor and the target molecule in an individual device that is multifunctional, manageable, and portable. Moreover, differently from traditional SERS substrates, D24 systems are sufficiently small to act as tracers. Released in the microcirculation circuits, D24 systems will be transported through the arteries, arterioles, and microvessels of the living tissue, interact with blood and the waste product of cells, internalize analytes, peptides and biomarkers, and operate analysis of biomolecules with elevated spatial and temporal resolution. Analytical maps of biomolecules may be, in turn, associated to individual cancer risk, pathological risk, or physiological state of a patient to support medical decisions and plan interventions.

Notice that the idea of using diatoms as microcapsules for sensing is not completely new. Nevertheless, previously reported works depart from our analysis to an extent that depends on individual contributions as explained in the subsequent section.

In reference [29], Ren and colleagues used simulations to explore the electric field enhancements generated by plasmonic nanoparticles coated on the surface of diatom skeletal shells. Then, they prepared SERS substrates by assembling silver nanoparticles on diatom surfaces. While similar devices achieve excellent sensing enhancement factors, diatoms are immobilized on a substrate, and may not be freely administrated in the microcirculation, within biological fluids or solutions, biological compartments, aqueducts, channels, seawater, ocean streams and currents, for biological or technical applications.

In reference [30], Chen and colleagues pressed Au nanoparticle-coated diatomaceous earth into hard, button-like millimeter tablets. Then, they used these SERS tablet devices to analyze the chemical composition of eccrine sweat in latent fingerprints, that is a brilliant, very practical application of the device in medicine. Yet, it is specific and the analysis is still executed at a macroscale level.

In reference [31], the group led by Luca De Stefano functionalized diatom frustule with Au nanoparticles using electroless deposition. Then, they tested the device using p-Mercaptoaniline (pMA). pMA can form self-assembled monolayer on metal surfaces and is therefore used as a surface probe molecule in SERS. Electroless deposition is an assessed technique for the synthesis of gold nanoparticles on an autocatalytic surface that allows attaining high control on the size and density of the particles [18, 19, 32]. Differently from this approach, we used here a photo-deposition process that is more direct, faster, and requires no or minimal sample treatment compared to electroless deposition. Nevertheless, the approach proposed by De Stefano is promising and deserves to be verified even further in biological or environmental applications.

## Conclusions

We developed ways to modify economic, easily accessible, and abundant diatomaceous earth to obtain miniature sensor devices where the pores of the diatom shells have the ability to capture molecules in solution, and the gold nanoparticles amplify the spectroscopy signal of several order of magnitude to reveal molecules in otherwise unattainable low abundance ranges. We demonstrated similar D24 devices in the analysis of biological BSA proteins in solution and for the detection of traces of mineral oil in a binary emulsion with water. In both cases, we revealed target molecules in low dilutions down to 10^−16^ M for BSA and 50 ppm for mineral oil. The devices may find applications in analytical chemistry, the surveillance and assessment of biological risk, food safety, contaminant monitoring, and surveillance in seawaters, aqueducts, and drinking water.

## Methods

### Scanning Electron Microscopy of Samples

Silica shells functionalized with Au nanoparticles (D24 systems) were dispersed directly on a carbon adhesive tape for scanning electron microscopy (SEM) imaging. Samples were imaged using a Zeiss Auriga Compact FE-SEM equipped with InLens secondary-electron detector, for morphological imaging, and anular Back Scattering detector, for Z-contrast imaging (in order to highlight the presence of Au).

### Characteristics of Diatomaceous Earth Used in This Study

Diatom material used in this study was a food grade, high-quality diatomaceous earth provided by Perma-Guard (Perma-Guard Europe Sollaris Sp. z o.o., Otwock, Poland) as a 1 kg free sample of Fossil Shell Flour®. Its current market price is ~ 16 euro per 1 kg. It is composed of cylindrical shells of extinct freshwater diatom Melosira preicelanica. Its main fraction constitutes amorphic silica (up to 94%), followed by smectites (~ 3%), kaolinite (~ 2%), feldspars (~ 1%), calcite (> 1%), and quartz (> 1%). Grinding in a low-speed hammer mill was performed to homogenize the particle size. Main properties (including physical and optical properties) of Fossil Shell Flour® are median particle size: 10 μm, mesh screen residue: 2%; refractive index: 1.43; oil absorption: 120%; brightness (green filter): 85; specific gravity: 2.2; surface area: 44.2 m^2^/g; pH: 8.0; total volume of pores: 0.132 cm^3^/g; micropores (< 20 Å): 14%; mesopores (20–500 Å): 65%.

### Fluorescence Analysis of D24 Systems

D24 systems were incubated with Fluoresbrite® Yellow Green Microspheres with a diameter of 50 nm for 10 min, with a ratio D24 : fluorescent particles = 1 : 10. Then, we collected D24 systems from the solution and placed them on the optical stage of an inverted Leica TCS-SP2® laser scanning confocal microscopy system. All measurements were performed using a ArUv laser. The pinhole (80 μm) and laser power (80% power) were maintained throughout each experiment. Yellow fluorescence (similar to FITC) was excited using a *λ*_1_ = 441 nm excitation line and confocal images were collected at the maximum of emission *λ*_2_ = 485 nm using × 10/20 objectives. Images were acquired over a region of interest of 975 × 750 μm^2^ and were averaged over four lines and ten frames to improve quality and reduce noise. Images were digitalized into 1280 × 960 pixels.

### X-Ray Photoelectron Spectroscopy Analysis of Samples

X-ray photoelectron spectra were recorded on a X-ray photoelectron spectroscopy (XPS) Versa Probe II (PHI, Chanassen US) by large area analysis mode where the mono-chromate Al anodic beam of 100 μm, 100 W power, normal to the surface, is rastered over an area of 1400 × 300 μm^2^ with the analyzer at 45° with respect to the sample surface. Survey spectra were acquired with an accumulation time at least of 20 min at high pass energy (187 keV) while high-resolution spectra of the elements of interests where acquired at 11.7 keV with same power and 0.1 eV resolution. Spectra were analyzed by Multipack (PHI, Chanassen USA) software and all the peaks were referenced to the adventious carbon peaks C1s at 284.8 eV binding energy.

### Simulating the Electromagnetic Field Within the D24 Systems

In order to calculate numerically the electric field profile throughout the decorated structure, a finite element method (FEM) 3D model has been developed employing the commercial software COMSOL Multiphysics 5.3. Simulations have been conducted on a single cubic unit cell where 125 particles have been placed on the surface of the patterned dielectric surface. The overall optical response has been investigated as a function of the incident angle of the electromagnetic field which was approximated as a TM linearly polarized plane wave (Additional file 4). The periodicity of the system was taken into account by applying Floquet boundary conditions on the lateral sides of the unit cell, perpendicular to the plane of incidence; subsequently, results have been periodically extended to visualize the diatom array (Additional file 4). A wavelength of *λ* = 633.0 nm was set. Power of the incident radiation was arbitrarily chosen as *P*_*inc*_ = 1 W, the unit cell area equals 3.9 × 10^−13^ m^2^ and the resulting intensity is *I* = 2.5 × 10^−8^ W/cm^2^ (notice that intensity dependant non-linearity is here neglected). Regarding the materials, the diatom was optically described as a dielectric with refractive index *n*_diatom_ = 1.3, whereas the surrounding environment is air with *n*_air_ = 1. Gold nanoparticles, modeled as perfect spheres with a diameter *d* = 20 nm, were modeled following the dielectric formulation reported in [20]. The geometrical domain has been discretized using tetrahedral elements. Maximum size of the mesh element has been chosen as 1/5 of the effective wavelength value that had to be resolved in each domain, depending on its refractive index. The minimum mesh element was set to *r*/1.5, *r* = 10 nm is the radius of each nano-sphere. Maxwell equations have been numerically solved within the unit cell by placing perfectly matched layers at the top and the bottom of the structure, in order to avoid unphysical reflections at the boundaries of the domain. In addition, the electromagnetic field symmetry has been exploited to reduce the computational effort of the simulation. As a result, equations are solved for a half of a diatom only, and perfect magnetic conductor boundary conditions have been imposed to the lateral sides of the unit cell, parallel to the plane of incidence, coherently with the polarization of the incident field.

### Raman Analysis of Samples

D24 devices containing BSA were positioned on the stage of a Renishaw inVia micro-Raman microscope for analysis. Samples were analyzed using × 20/50 objectives of a Leica microscope. Raman spectra were excited by the 633.0 nm line of an HeNe laser in backscattering geometry and acquired with a CCD with 1024 × 1024 pixels. Laser power was adjusted as 0.18 mW and maintained constant throughout the whole measurements. Interferograms were recorded with an integration time of 20 s. Each spectrum was base line corrected with a second degree polynomial function. Raman maps were performed with a step size of 400 and 600 nm in the *x* and *y* axes direction.

## Additional Files

Additional file 1: SEM images of D24 systems. (DOCX 878 kb)

Additional file 2: Notes on the *diatomaceous earth* used in this study. (DOCX 18 kb)

Additional file 3: Fluorescence images of D24 systems. (DOCX 515 kb)

Additional file 4: Supporting figures to the Numerical Simulation Methods of the main text. (DOCX 608 kb)

## Abbreviations

BSA: Bovine serum albumin
D24 systems: Silicon dioxide diatom shells functionalized with gold nanoparticles
DE: Diatomaceous earth
MO: Mineral oil
SERS: Surface-enhanced Raman spectroscopy
