# Supplementary material for: Silica diatom shells tailored with Au nanoparticles enable sensitive analysis of molecules for biological, safety and environment applications
Source: Nanoscale Res Lett. 2018 Apr 10;13:94. doi: 10.1186/s11671-018-2507-4 (PMC5891442; doi:10.1186/s11671-018-2507-4)
Supplement: Supplementary file 1 — SEM images of D24 systems. (DOCX 878 kb) [file 11671_2018_2507_MOESM1_ESM.docx]

**Additional file 1. SEM images of** $\mathbf{D24}$ **systems.**

SEM images of $D24$ systems are reported in the **Supporting Figures 1.1** and **1.2**. High magnification factor SEM images in the **Supporting Figures 1.3** show that gold nanoparticles are distributed either on the external surface of diatoms and deep within the pores.


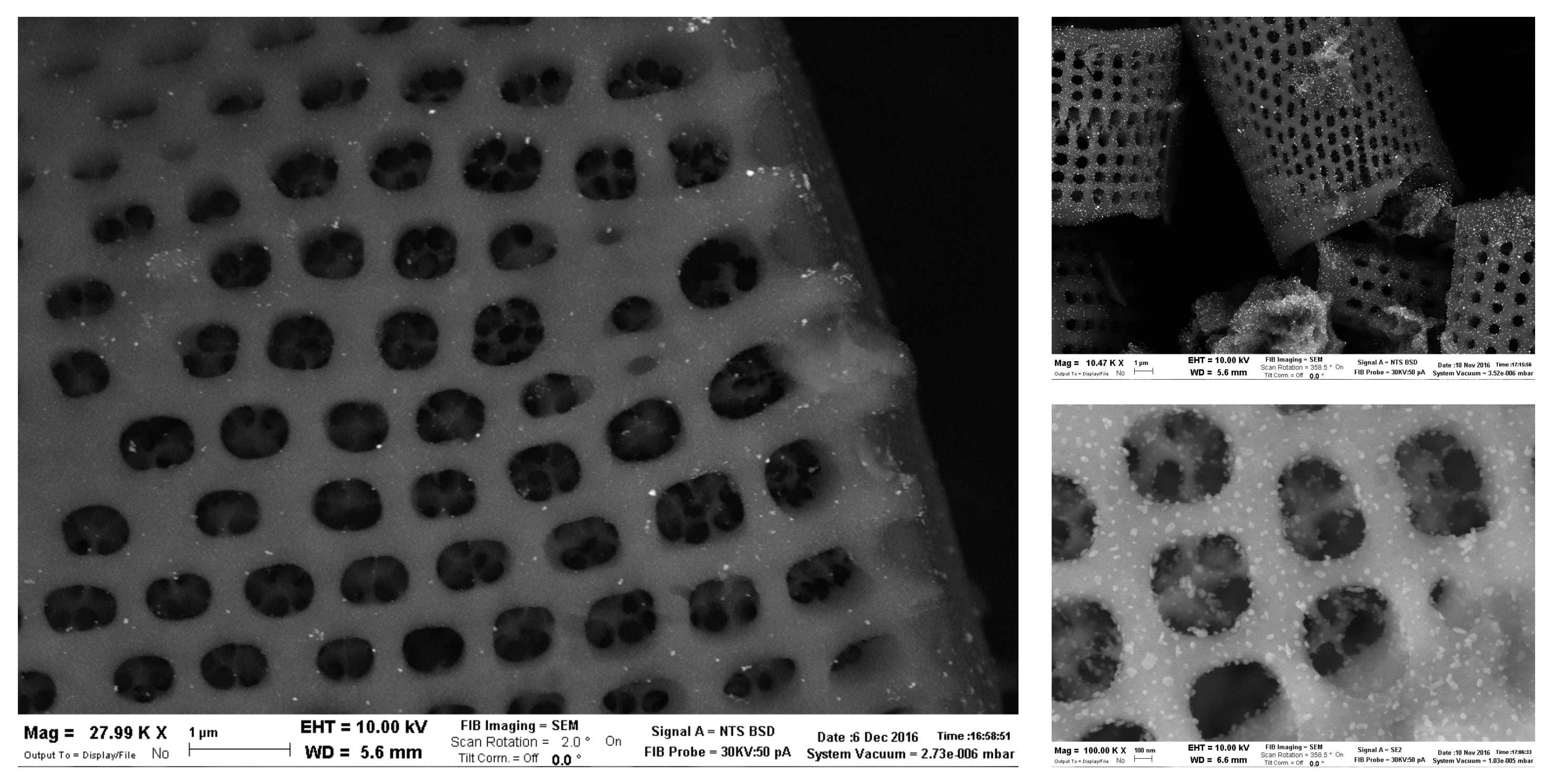


**Supporting Figure 1.2**


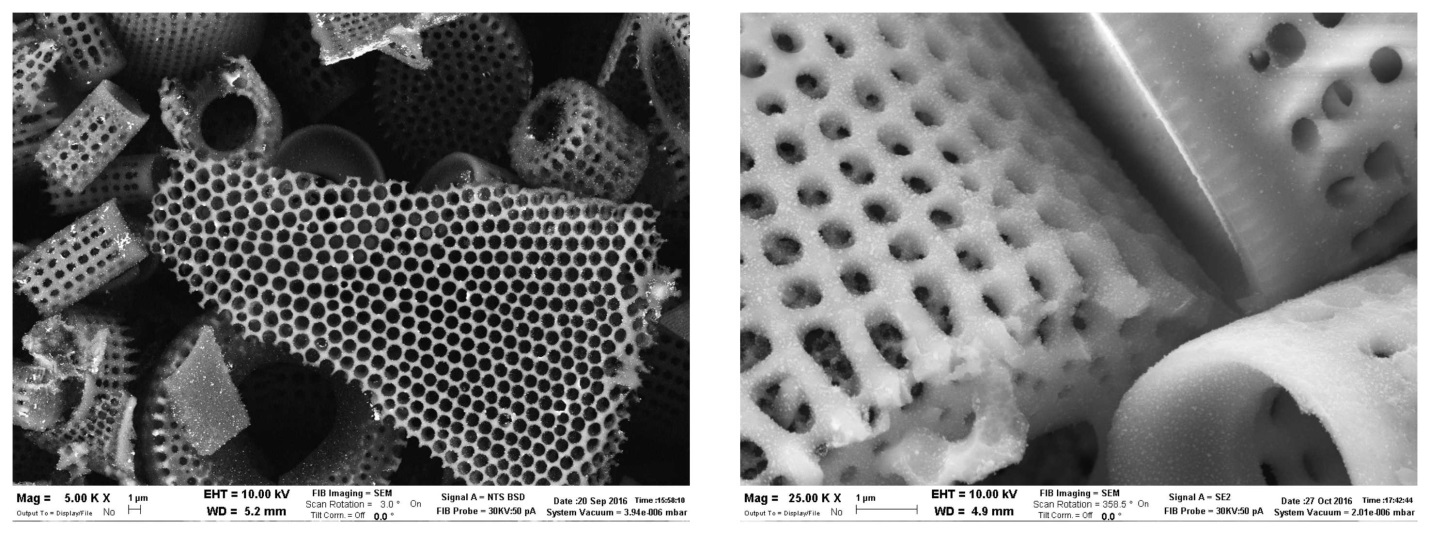


**Supporting Figure 1.2**

**
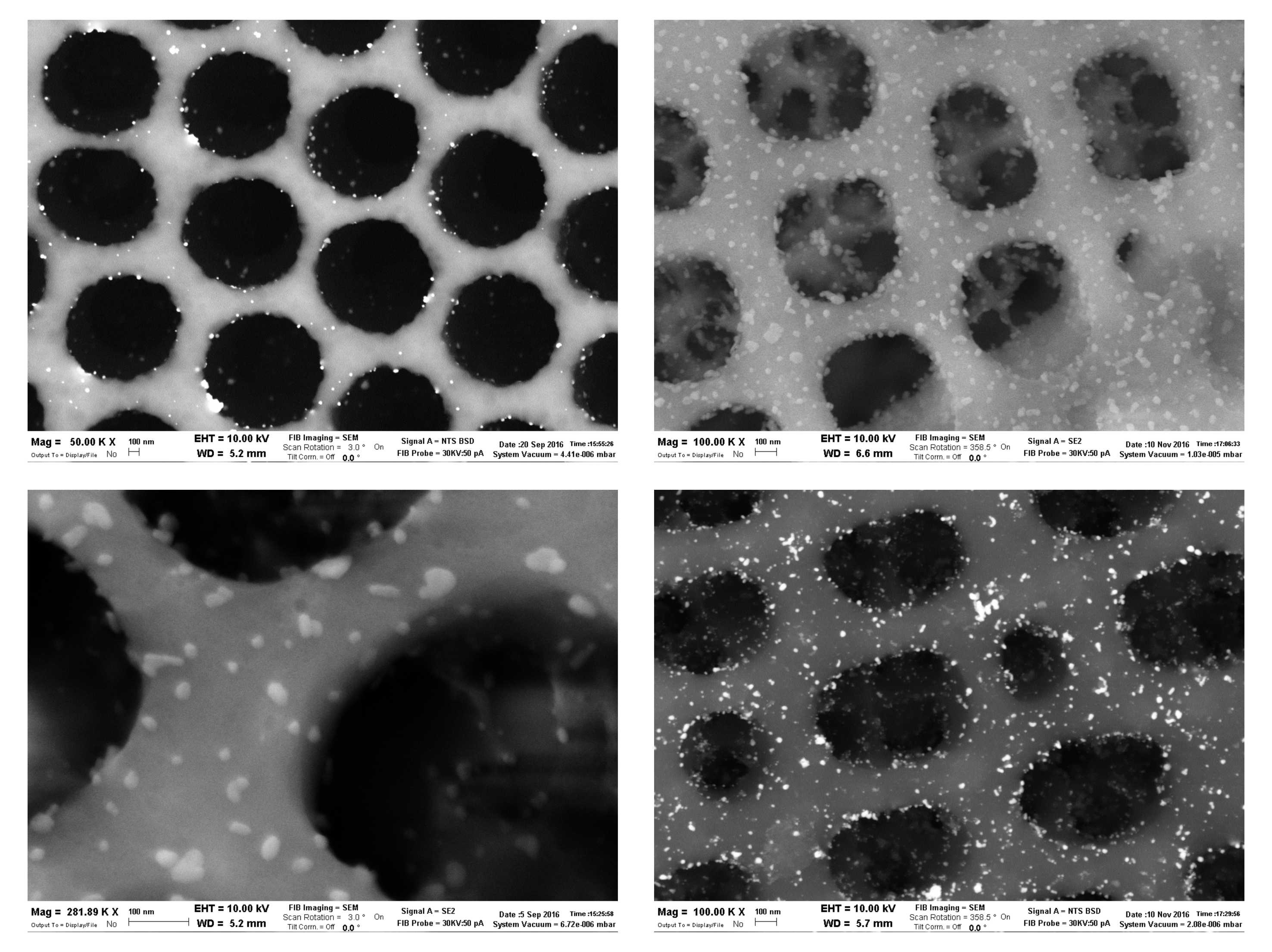
**

**Supporting Figure 1.3**
