# Supplementary material for: Silica diatom shells tailored with Au nanoparticles enable sensitive analysis of molecules for biological, safety and environment applications
Source: Nanoscale Res Lett. 2018 Apr 10;13:94. doi: 10.1186/s11671-018-2507-4 (PMC5891442; doi:10.1186/s11671-018-2507-4)
Supplement: Supplementary file 2 — Notes on the diatomaceous earth used in this study. (DOCX 18 kb) [file 11671_2018_2507_MOESM2_ESM.docx]

**Additional file 2. Notes on the *diatomaceous earth* used in this study.**

Diatoms, unicellular algae occupying almost every aquatic and semi-aquatic niche worldwide, contribute up to of both oceanic primary production and global oxygen production. One of the most intriguing and evolutionary important features of diatoms is undoubtedly their hard siliceous cell wall called frustule. Every diatom frustule is richly ornamented with species-specific symmetrical rows of thickenings and pores of various sizes, shapes, and structural complexity. Currently, diatom species richness is estimated to be more than species with possibly as many different frustules bearing unique combinations of ultra-structurally simple and complex perforations.

Diatomaceous earth, also known as diatomite, is a light, porous sedimentary rock formed from diatom shells that accumulated in the areas of high diatom production and sedimentation. Its main component is amorphous opaline silica () that is partially transformed into anhydrous silica in older sediments. Due to the unique characteristics of diatom skeletons, diatomite has been used as a building and filtrating material since the ancient times. With rapid technological developments and constant innovation in various sectors of applied science, diatomaceous earth and siliceous parts of modern diatoms start receiving more widespread attention. Advantages and disadvantages of using diatom shells from both diatomite and diatom cultures in applied sciences are summarized in the **Supporting Information Table 2.1**.

|  | Diatomite | Diatom cultures |
| --- | --- | --- |
| **price** | *cheap* | *relatively cheap/moderately expensive*  - many diatom species can be successfully cultivated in laboratories equipped with basic facilities and instruments, in room temperature and semi-sterile conditions. However, other species may require appropriate medium, light conditions, oncentration, temperature etc. to proliferate;  - acids or other concentrated aggressive agents (e.g. hydrogen peroxide) have to be used to digest the organic parts of the cells and clean the frustules; |
| **time** | *instantly available in large quantities* | *slow growth in culture*  - obtaining several g of cleaned material in standard diatom lab may take months or more (depending on the diatom species); algal bioreactors may be used to increase the efficiency |
| **quality** | *low/moderate*  - silica in old deposits is progressively transformed into porcellanite (anhydrous silica) and finally to quartz (significant decrease in porosity)  - diatomite contains a relatively high number of broken frustules (irregular size and shapes of the particles)  - may contain other minerals | *very high*  - material obtained from diatoms cultivated in controlled conditions will contain up to of silica;  - appropriate cleaning procedure will largely reduce the number of broken frustules (highly similar particle shape and size); |
| **diatom diversity** | *very low*  - due to various silica content and external conditions, very few diatom species remain preserved in old sediments (very limited pool of forms and pore sizes);  - diatomite species composition cannot be modified; | *very high*  - potentially any diatom species can be cultured and harvested (very high number of available forms and pore sizes);  - cleaned frustules of different species can be mixed in various proportions; |
| **future prospects** | *limited*  - new diatomite deposits are forming (a few other diatom species will possibly be found in sediments from various region) | *genome manipulations*  - ongoing studies has already indicated genes and regions involved in silica bioprocess (=genetic manipulations may allow designing and fabrication of desirable siliceous forms and perforations). |

**Supporting Information Table 2.1.** Advantages and disadvantages of using diatom shells from both diatomite and diatom cultures in applied sciences.
