# Supplementary material for: Silica diatom shells tailored with Au nanoparticles enable sensitive analysis of molecules for biological, safety and environment applications
Source: Nanoscale Res Lett. 2018 Apr 10;13:94. doi: 10.1186/s11671-018-2507-4 (PMC5891442; doi:10.1186/s11671-018-2507-4)
Supplement: Supplementary file 3 — Fluorescence images of D24 systems. (DOCX 515 kb) [file 11671_2018_2507_MOESM3_ESM.docx]

**Additional file 3. Fluorescence images of systems.**

Fluorescence images of systems are reported in the **Supporting Figures 3.1** and **3.2**. Fluorescence images of systems in the **Supporting Figure 3.3** demonstrate spatial overlap between the fluorescence signal and the D24 diatom devices. This evidence along with the vanishingly small background signal demonstrate co-localization between the analyte and the diatom pores: in this process, analyte uptake is highly efficient and leves no or minimal residues.

|  |  |
| --- | --- |
| Supporting Figure 3.1 | **Supporting Figure 3.2** |

**Supporting Figure 3.3**
