# Supplementary material for: Silica diatom shells tailored with Au nanoparticles enable sensitive analysis of molecules for biological, safety and environment applications
Source: Nanoscale Res Lett. 2018 Apr 10;13:94. doi: 10.1186/s11671-018-2507-4 (PMC5891442; doi:10.1186/s11671-018-2507-4)
Supplement: Supplementary file 4 — Supporting figures to the Numerical Simulation Methods of the main text. (DOCX 608 kb) [file 11671_2018_2507_MOESM4_ESM.docx]

**Additional file 4. Supporting figures to the Numerical Simulation Methods of the main text.**

The optical response of the system has been investigated as a function of the incident angle of the incident electromagnetic field (**Supporting Figure 4.1**). The periodicity of the system was taken into account by applying Floquet boundary conditions on the lateral sides of the unit cell; results have been periodically extended to visualize the diatom array (**Supporting Figures 4.2-4.3**). In the three-dimensional scheme of the **Supporting Figure 4.4**, gold nanoparticles are distributed along the internal surface of the pores. Thus FEM simulations recover the EM field distribution around the gold nanoparticle clusters and within the pore matrix where the analytes are retained.

| 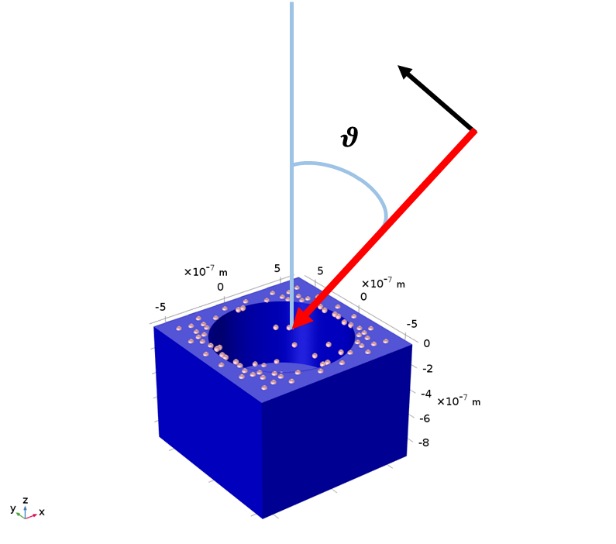 | 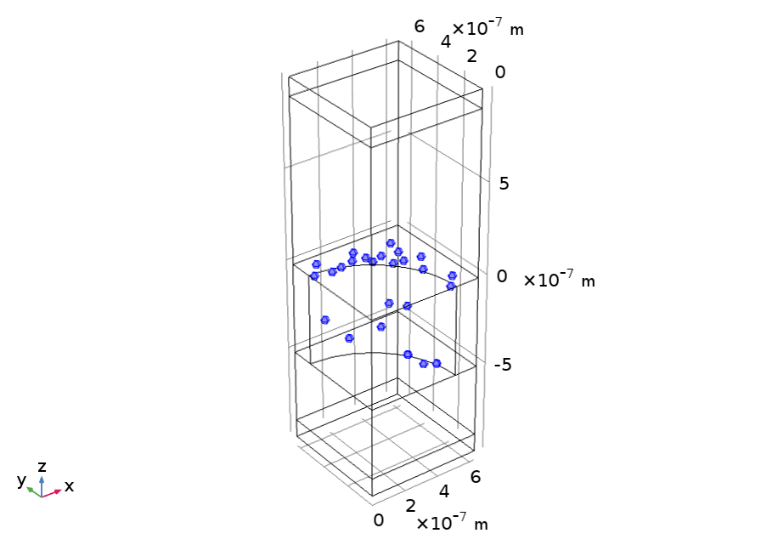 |
| --- | --- |
| Supporting Figure 4.1 | **Supporting Figure 4.2** |


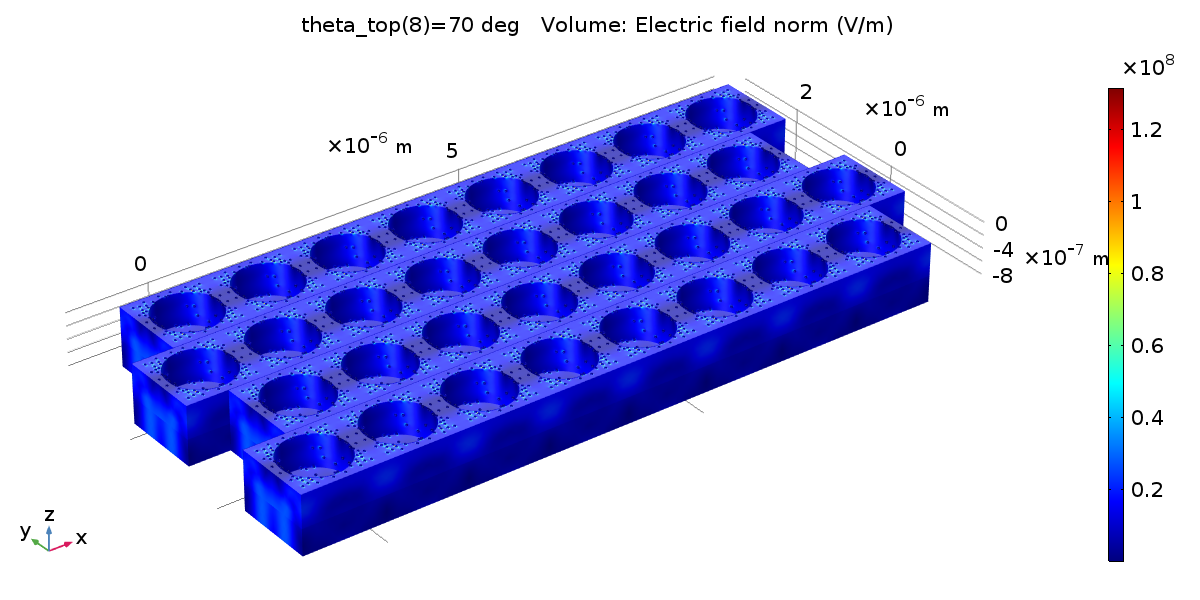


**Supporting Figure 4.3**

**
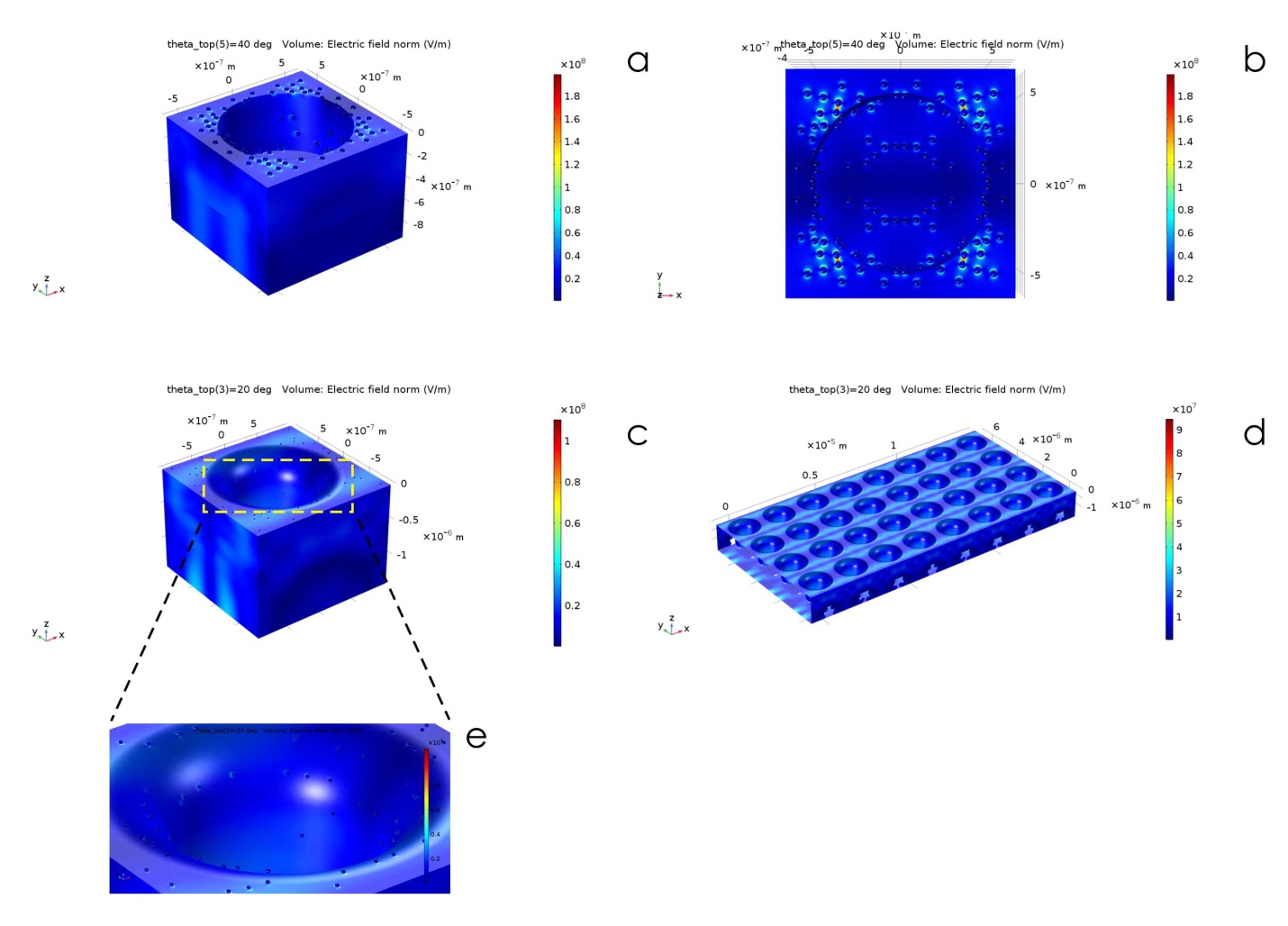
**

**Supporting Figure 4.4**
